# Safety Profile and Complication Rates in Emergency Off-label Use of Tirofiban in Interventional Neuroradiology: An Observational Dual Center Study

**DOI:** 10.1007/s00062-022-01223-5

**Published:** 2022-10-21

**Authors:** Carolin Brockmann, Daniel Dillinger, Anastasios Mpotsaris, Annette Spreer, Volker Maus, Stephan Waldeck, Ahmed E. Othman, Sebastian Altmann, Florian Ringel, Thomas Kerz, Marc A. Brockmann

**Affiliations:** 1 Department of Neuroradiology, University Medical Center Mainz, Langenbeckstr. 1, 55131 Mainz, Germany; 2 Department of Radiology and Neuroradiology, Bundeswehr Central Hospital Koblenz, Rübenacher Str. 170, 56072 Koblenz, Germany; 3 Department of Neuroradiology, Munich Klinik, Thalkirchner Str. 48, 80337 Munich, Germany; 4 Department of Neurology, Klinikum Braunschweig, Salzdahlumer Straße 90, 38126 Braunschweig, Germany; 5 Department of Neurology, University Medical Center Mainz, Langenbeckstr. 1, 55131 Mainz, Germany; 6 Department of Radiology, Neuroradiology and Nuclear Medicine, Universitätsklinikum Knappschaftskrankenhaus Bochum, In der Schornau 23–25, 44892 Bochum, Germany; 7 Department of Neurosurgery, University Medical Center Mainz, Langenbeckstr. 1, 55131 Mainz, Germany

**Keywords:** Emergency treatment, Platelet inhibtion, Platelet aggregation, Side effect, Hemorrhage

## Abstract

**Purpose:**

Tirofiban has been approved for the treatment of acute coronary syndrome. Meanwhile, tirofiban is frequently applied in emergency situations in interventional neuroradiology (INR). The objective of this study was to analyze the risk profile for the off-label use of tirofiban in INR patients.

**Methods:**

Data of 86 patients, who underwent neurointerventional therapy and were treated with tirofiban at 2 neuroendovascular centers between January 2016 and July 2017 were retrospectively analyzed. Despite off-label use, recent stroke (< 30 days), recent hemorrhage, thrombocytopenia (< 150,000/µl), activated partial thromboplastin time (aPTT) > 1.3-fold, internation normalised ratio (INR) < 1.5, severe liver insufficiency (Child-Pugh C), and preceding intravenous thrombolysis were considered as contraindications.

**Results:**

Median patient age was 62 years (range 26–88 years). Patients received tirofiban for extracranial (*n* = 35) or intracranial stenting (*n* = 35), coiling of ruptured cerebral aneurysms (*n* = 6), continuous intra-arterial nimodipine infusion via microcatheters for subarachnoid hemorrhage (SAH)-related vasospasm (*n* = 5), or thrombotic complications during neuroendovascular procedures (*n* = 5). The desired effect of preventing thrombotic complications when applying tirofiban off-label was achieved in 81 of 86 patients (94.2%). Relevant tirofiban-associated complications occurred in 14 patients (16.3%), of which 9 patients received i.v. thrombolysis for treatment of acute ischemic stroke shortly before starting therapy with tirofiban. Of the 86 patients 12 died, while the overall tirofiban-related mortality was 2.3% (2 patients died due to ICH). Logistic regression analysis revealed age to be the only parameter significantly associated with development of tirofiban-associated complications (*p* = 0.026).

**Conclusion:**

Whereas the safety profile of tirofiban when applied off-label in INR is acceptable, the highest risk for relevant tirofiban-associated complications is observed in older patients treated by emergency stenting for acute stroke.

## Background

The need for immediate and potent platelet inhibition is a frequently encountered scenario in modern interventional neuroradiology (INR). Acute platelet inhibition may be required in settings such as emergency extra- or intracranial stenting including the implantation of flow diverting stents, the management of periprocedural thromboembolic complications or the treatment of large vessel vasospasm by continuous intra-arterial infusion of vasodilating drugs via microcatheters placed in the internal carotid artery (ICA) up to several days [1].

Whereas different glycoprotein (GP) IIb/IIIa receptor inhibitors are available [2, 3], one of the most frequently used drugs in INR is the non-peptide selective glycoprotein GP IIb/IIIa receptor inhibitor tirofiban. Tirofiban reversibly inhibits fibrinogen-dependent platelet aggregation and subsequent formation of thrombi. Due to its high receptor affinity and short plasma half-life, it is even capable of dissolving fresh thrombi, thus offering a high potential for platelet inhibition in the aforementioned situations.

Tirofiban has been proven to be safe and effective in the prevention of acute myocardial infarction without ST-elevation in patients with acute coronary syndrome [4, 5]. Furthermore, tirofiban is frequently applied in critically ill patients with one or more contraindications normally excluding the use of tirofiban, such as freshly surgically treated patients (e.g. aneurysm clipping or insertion of an external ventricular drain) or patients who just underwent i.v. thrombolysis. From a medicolegal point of view in these patients the use of tirofiban would be contraindicated. Therefore only a few case series describe the off-label use of tirofiban in neuroendovascular interventions [6]. The risk of peri- or postprocedural intracranial hemorrhage (ICH) using tirofiban in patients with ischemic stroke has been reported to range between 2.7% and 33% [7–11].

Considering the lack of evidence for the frequent off-label use of tirofiban in INR, the underlying study aimed at analyzing the efficacy and the risk for peri- or postprocedural tirofiban-associated complications with a special focus on neuroendovascular patients with contraindications for tirofiban.

## Material and Methods

The IRB waived the requirement to obtain a signed consent form due to the retrospective nature of this study.

Between January 2016 and July 2017, 1345 endovascular, neurointerventional procedures were carried out at 2 neuroendovascular centers. Data of 86 consecutive patients who underwent neurointerventional treatment and were treated with tirofiban during and in some cases after the procedure were identified. Tirofiban was administered intravenously starting with 0.4 µg/kg body weight/min for 30 min and afterwards maintained at 0.1 µg/kg/min for as long as required. The choice of neuroendovascular devices was left to the discretion of the neurointerventionalist. Patient outcome with a special focus on hemorrhagic complications was analyzed.

Hemorrhage was considered tirofiban-related if it occurred between the beginning of tirofiban infusion and up to 10 h after ending treatment with tirofiban. Hemorrhagic complications included all kinds of ICH (i.e. hemorrhagic infarction and parenchymal hematoma [12]), gastrointestinal bleeding and macrohematuria as well as any other relevant hemorrhage. Development of pseudoaneurysm of the groin was also considered to be a tirofiban-related complication.

The evaluation focused on the relation between contraindications as listed in the tirofiban package insert and the occurrence of hemorrhagic complications. According to the package insert, we considered contraindications to be recent stroke (< 30 days), recent hemorrhage (especially ICH), thrombocytopenia (< 100,000/µl), activated partial thromboplastin time (aPTT) > 1.3-fold, INR < 1.5, severe liver insufficiency (Child-Pugh C) and intravenous thrombolysis treatment for acute stroke (combined contraindication).

If available, efficacy of tirofiban was documented in digital subtraction angiograms (DSA). Thus, successful thrombolysis using tirofiban, the lack of new thrombotic complications and in-stent thrombosis were considered as successful use of tirofiban. In cases without postinterventional DSA, we analyzed CT-angiography, magnetic resonance angiography or sonographic examinations to control the success. The absence of postprocedural diffusion restriction in diffusion-weighted magnetic resonance imaging was also considered as success.

### Statistical Analyses

Retrospective descriptive statistical evaluation was performed using SPSS 23.0 (IBM, Armonk, NY, USA). A 5% significance level was assumed. Subgroup analysis regarding contraindications and treatment site were performed. A logistic regression model was applied to evaluate the relation between contraindications and the occurrence of hemorrhagic complications.

## Results

### Patients

We identified 86 patients treated with tirofiban during or after neuroendovascular procedures between January 2016 and July 2017. Of the patients 56 (65.1%) were treated at center 1, and 30 (34.9%) patients were treated at center 2. Median patient age was 62 years (range 26–88 years), 38 patients were female (44.2%), 48 patients were male (55.8%). Median duration of tirofiban infusion was 23 h (range 0.5–381 h). Median platelet count decreased by 10,000/µl (range 135,000–165,000/µl; *p* = 0.841) under therapy with tirofiban. Lowest observed platelet count was 90,000/µl. Tirofiban effectively prevented or resolved thromboembolic complications in 81 out of 86 cases (94.2%). In 4 patients (4.7%) tirofiban failed to prevent thromboembolism and 1 patient (1.2%) was transferred to another hospital and therefore lost to follow-up.

Thirty-five patients (40.7%) were treated by intracranial stenting, whereas another 35 patients (40.7%) underwent extracranial stent PTA. Six patients (7%) suffered from ruptured cerebral aneurysm and were treated by coil embolization (without stenting). Five patients (5.8%) with SAH-related large vessel vasospasm (due to rupture of a cerebral artery aneurysm) were treated with continuous intra-arterial nimodipine infusion with intra-arterial microcatheter placement. Another 5 patients (5.8%) were treated with tirofiban for peri-interventional thrombus formation during other neuroendovascular procedures.

**Fig. 1:**
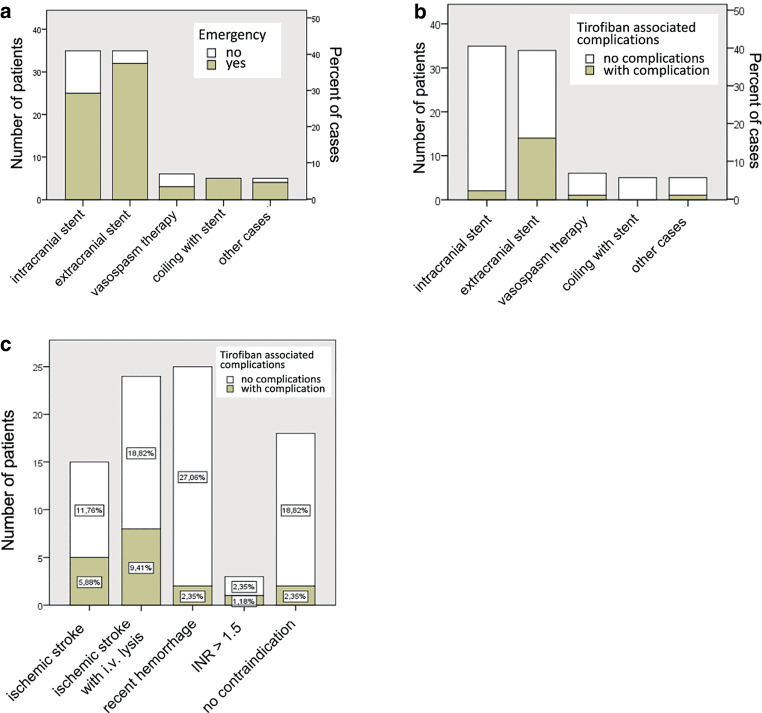
**a** Absolute and relative numbers of different procedures performed using tirofiban in an elective and emergency setting. **b** Distribution of tirofiban associated complications during the different endovascular procedures. **c** Distribution of tirofiban-associated complications depending on the type of contraindication. *INR* international normalised ratio

Of the 86 patients, 69 (80.2%) were rated emergency cases, and 17 patients (19.8%) underwent elective treatment. Fig. 1a provides an overview of patient distribution within these different treatment groups and indicates the type of treatment (elective vs. emergency treatment).

Despite the fact that tirofiban is frequently applied off-label in INR, one or more contraindications for tirofiban existed in 67 patients (77.9%). These were acute or subacute stroke (as verified by MRI or CT) in 45 patients (52.3%), recent i.v. administration of thrombolysis (rtPA) for acute stroke (*n* = 28; 32.6%), and treatment with anticoagulants in 15 patients (17.4%). One patient suffered from severe kidney insufficiency (in this case tirofiban dosage was not reduced as suggested by the vendor) and 1 patient presented with severe liver insufficiency (Child-Pugh C). Six patients (7%) presented with a platelet count below 150,000/µl at the time of starting therapy with tirofiban.

### Tirofiban-associated Complications

Overall, hemorrhagic complications were observed in 18 out of 86 patients (20.9%) with 14 patients (16.3%) developing any kind of ICH. One patient developed macrohematuria, 2 patients suffered from gastrointestinal bleeding, and 1 patient developed a pseudoaneurysm of the groin, which we also decided to be attributable to impaired hemostasis.

All in all, 12 of 86 patients (14.0%) receiving tirofiban died. Of these, however, only 2 patients (2 of 86; 2.3%) died due to possibly tirofiban-related ICH after emergency stent PTA. One of the 2 patients who died due to a tirofiban-related ICH also received additional i.v. thrombolysis. Ten patients died due to other complications related to acute stroke and subarachnoid hemorrhage. An overview of the number of complications with regard to pre-existing contraindications or performed procedure is provided in Fig. 1b, c.

In the group of the 28 stroke patients receiving i.v. thrombolysis before initiation of treatment with tirofiban, 9 patients (32.1%) developed a tirofiban-related complication and 3 (10.7%) of the 28 patients receiving i.v. thrombolysis died.

The complication rate in patients with a contraindication for tirofiban was significantly higher (23.9%) compared to the group without contraindications for treatment with tirofiban (11.1%) (*p* < 0.05; Table 1).

**Table 1:** Interaction between contraindications and complications in tirofiban treatment

			Complications		Total
			No tirofiban-related complications	Tirofiban-related complications	
Contraindications	Without contraindications	Number	16	2	18
			88.9%	11.1%	100.0%
	With contraindications	Number	51	16	67
			76.1%	23.9%	100.0%
Total		Number	67	18	85
			78.8%	21.2%	100.0%

Only 1 of the electively treated patients (who received tirofiban to solve any procedure-related thrombotic complication) developed a tirofiban-associated hemorrhage, whereas 17 patients of the emergency treatment group developed any probably tirofiban-associated hemorrhage. Due to the small number of elective patients with tirofiban-associated complications, however, we were not able to detect a significant difference between both groups (*p* = 0.105).

Subgroup analysis showed the highest complication rates in patient with acute ischemic infarction and acute ischemic infarction with preceding i.v. thrombolysis. We found age to be the only parameter significantly increasing the risk to develop a possibly tirofiban-associated complication (*p* =0.026; Table 2). Per life year, the risk for a tirofiban-related complication increased by 7.2%.

**Table 2:** Logistic regression analysis adjusted for institution, patient age and gender

	Regression-coefficient B	Standard error	*p*-value	Exp(B)	95% confidence interval for Exp(B)	
					Lower	Upper
Application duration	0.000	0.011	0.993	1.000	0.980	1.021
Emergency procedure	1.701	1.506	0.259	5.478	0.286	104.867
Contraindications	−0.146	1.214	0.905	0.865	0.080	9.333
Institution	0.043	0.668	0.948	1.044	0.282	3.864
Age	0.070	0.026	0.007*	1.072	1.020	1.127
Gender	−0.451	0.608	0.458	0.637	0.194	2.095
Constant	−6.944	2.442	0.004	0.001	–	–

*Exp* exponential value of B, odds ratio

*indicates statistical significance

## Discussion

Due to its high efficacy tirofiban is frequently used to achieve platelet inhibition in INR [13, 14]. Several conditions, such as preceding ischemic and hemorrhagic stroke during the past 30 days, low platelet count, other precedent bleedings and more are considered to be a contraindication for the use of tirofiban. Relative contraindications include low creatinine clearance, liver insufficiency and low platelet count. Furthermore, the concomitant use of heparin, warfarin, and other novel oral anticoagulants or thrombolytics may increase the risk for (intracranial) hemorrhage. In a non-INR setting, the risk for ICH under treatment with tirofiban has been reported to range between 2.2% and 0.1%. Thrombocytopenia below 90,000/µl was reported in 1.5% of patients being treated with tirofiban and heparin, whereas severe thrombocytopenia below 50,000/µl was observed in 0.3% [15].

Although frequently applied, only few case series analyzed the incidence of hemorrhagic complications resulting from off-label use of tirofiban in neuroradiological interventions. The overall complication rate reported in the literature using tirofiban in neuroendovascular treatment ranged between 0% and 33.0% [6–12, 16–20]. The safety of tirofiban in acute ischemic stroke (SaTIS) study group analyzed complication rates in patients with acute stroke and the need for intravenous administration of tirofiban. Whereas the authors reported ICH in up to 30% of patients, the complication rate of the placebo group reached 26.6%. This matches well with our findings, which showed that of the 28 patients receiving i.v. thrombolysis in combination with tirofiban, 9 patients (32.1%) developed a complication.

In a smaller neurointerventional case series by Mangiafico et al. [18], 21 patients were treated for acute intracranial vessel occlusion by mechanical recanalization. All patients received tirofiban, heparin and intra-arterial thrombolysis. Of these patients, 5 developed ICH, with 2 of the 5 patients being related to periprocedural vessel injury, leaving 3 out of 21 patients (14.3%) with probably tirofiban-related ICH.

Regarding the use of tirofiban in patients undergoing surgical procedures, another interesting study was published by Bruder et al. [21]. They analyzed data of 444 patients requiring ventriculostomy following aneurysmal SAH. Of these, 117 patients treated endovascularly also received antiplatelet therapy, and 23 (20%) received tirofiban. Whereas ICH occurred significantly less often in patients without any antiplatelet therapy after ventriculostomy (8% vs. 24%; *p* < 0.05), patients treated with tirofiban developed ICH after ventriculostomy in up to 39%, although all of the hemorrhages remained without relevant symptoms. Since in our case series ventriculostomy was performed prior to endovascular treatment, we did not observe this kind of complication.

Another retrospective multicenter analysis focused on patients undergoing stent PTA for combined intracranial and extracranial stenosis. One center used tirofiban, whereas the other 3 centers used eptifibatid or ASA together with clopidogrel in these patients. In the tirofiban group 11% developed hemorrhage (7% symptomatic) compared to 9% of all patients without significant group differences [16].

The complication rate in our study was 20.9% and herewith well within the range of the results reported in the literature. Compared to the SaTIS study (which excluded patients with i.v. thrombolysis), our hemorrhage rate was lower despite the inclusion of patients with i.v thrombolysis. Tirofiban-related mortality rate of 2.3% in our study ranges in the lower level of the reported results in the literature [6–12, 16–20]. The rate of hemorrhage of 10.7% when combining tirofiban and i.v. thrombolysis in our results matches the data in the literature with 12% [8, 9]. Out of the 28 patients receiving both tirofiban and i.v. thrombolysis, 3 patients died but only 1 death was associated with tirofiban.

Although the risk for tirofiban-related complications was not significantly higher in the emergency group, there was an increased complication rate in emergency neuroendovascular treatment with tirofiban (6.3% vs. 24.6%). The highest rate of parenchymal hematoma was found in patients with i.v. thrombolysis for acute cerebral ischemia and a need for emergency extracranial stent PTA. In contrast to other studies [15, 16], the only associated risk factor in our study was age. Therefore, higher age, especially an age over 80 years, should seriously be taken into account before starting treatment with tirofiban.

Limitations of the study are its retrospective character and a selection bias. Although the sample size with 86 patients from 2 centers is quite small, it allowed systematic analyses, which in turn could generate hypotheses for further larger clinical trials. Our series does not focus on a single entity (e.g. acute stroke or stent PTA), but features a broad spectrum of indications representing a real-life setting. Furthermore, we did not focus on the underlying pathology, but on treatment with a specific drug, which we found to be of high interest from a medicolegal perspective, as we focused on the complication rate of a given drug which is frequently used despite contraindications. Finally, we were not able to clarify which of the observed complications are really due to treatment with tirofiban, as hemorrhagic complications also occurred even without additional platelet inhibition in stroke patients. Therefore, the rate of tirofiban-related complications (when applied off-label) might be even lower than reported in the underlying work.

Frequently, the risk of thrombotic complications must be balanced against the risk of hemorrhagic complications, especially in situations such as stroke with subsequent thrombectomy and stenting of a stenotic vessel, when the risk of intracranial hemorrhage is increased [22]. Therefore, several ways to reduce the risk of hemorrhagic complications while maintaining effective platelet inhibition have been proposed. Lin et al. compared the efficacy and safety between standard and low-dose tirofiban (36–48 h infusion of 0.10 μg/kg/min vs. 0.075 μg/kg/min) in the treatment of older high-risk non-ST-segment elevation acute coronary syndrome patients (*n* = 94) who underwent percutaneous coronary intervention (PCI) [23]. Success of PCI and rate of major adverse cardiac events were similar between the two groups, but major bleeding events were significantly higher in the standard dose group (10.4% vs 0.0%, *p* = 0.03).

Similarly, Li et al. compared 78 patients with non-ST elevation acute coronary syndrome undergoing PCI treated with a standard dose of tirofiban with a group of 85 patients treated with half-dose tirofiban (i.e. bolus dose of 10 or 5 μg/kg within 3 min followed by continuous i.v. infusion of 0.15 or 0.075 μg/kg/min for 48 h) [24]. They observed no significant differences in major adverse cardiac events as well major bleeding complications (no major hemorrhage in both groups), but only the incidence of minor bleeding was significantly lower in the half dose (HD) group compared to the standard dose (SD) group (8.2% vs. 20.5%, *p* = 0.04). Survival between both groups was comparable. Although it remains unclear to what extent these results can be transferred to a neuroradiological setting with patients suffering from acute stroke, similar studies in the field of neuroradiology are of interest.

Another way to reduce the risk of intracerebral hemorrhage in older patients with acute stroke and the requirement for acute stenting might be loading with 500 mg of acetylsalicylic acid (ASA) only (and dual platelet inhibition after 24 h), which is less aggressive regarding platelet inhibition and harbors the disadvantage of a prolonged half-life due to irreversible platelet inhibition. Recently, Pop et al. compared conservative (ASA only) with aggressive dual anti-platelet strategy for emergency carotid stenting during stroke thrombectomy [25]. They reported the aggressive periprocedural antiplatelet strategy to be associated with improved carotid stent patency, higher proportion of moderate clinical outcome, and found no significant differences in mortality and hemorrhagic transformation rates. Yet another possibility might be to terminate the infusion of tirofiban right after applying the loading dose, and to resume treatment with tirofiban 6–9 h later after ruling out relevant intracerebral hemorrhage by CT. During this time platelet inhibition is still effective. This regimen, however, requires further evaluation. In any case, rigorous blood pressure management following revascularization is mandatory in order to prevent hyperperfusion and to reduce the risk of hemorrhage, especially in cases of a damaged blood-brain barrier.

## Conclusion

We conclude that the safety profile of tirofiban when being used off-label in a neuroendovascular setting in principle is acceptable but point out that the proportionally highest complication risk applies to older patients and patients being treated for acute stroke. In these patient groups alternative concepts for platelet inhibition should be evaluated.
